# SULF1 regulates malignant progression of colorectal cancer by modulating ARSH via FAK/PI3K/AKT/mTOR signaling

**DOI:** 10.1186/s12935-024-03383-5

**Published:** 2024-06-06

**Authors:** Wenjie Zhu, Changlei Wu, Zitao Liu, Shimin Zhao, Xiufeng Cheng, Jun Huang

**Affiliations:** 1 Department of Gastrointestinal Surgery, The Second Affiliated Hospital, Jiangxi Medical College, Nanchang University, Nanchang, Jiangxi Province China; 2 Jiangxi Province Key Laboratory of Molecular Medicine, The Second Affiliated Hospital, Jiangxi Medical College, Nanchang University, Nanchang, Jiangxi Province China; 3 Department of Critical Care Medicine, The First Affiliated Hospital, Jiangxi Medical College, Nanchang University, Nanchang, Jiangxi Province China

**Keywords:** SULF1, ARSH, Colorectal cancer, FAK/PI3K/AKT/mTOR, Proliferation, Invasion, Metastasis

## Abstract

**Background:**

Colorectal cancer (CRC) has the third highest incidence and second mortality rate of malignant tumors globally, highlighting the urgency to explore the mechanisms underlying CRC progression for refined treatment of this patient population.

**Methods:**

R Studio was used for data sorting and analysis. Cell apoptosis and cell cycle detection were performed by flow cytometry. Quantitative real-time PCR (qRT-PCR) was used to explore mRNA expression levels. Western blotting was used to explore protein expression levels. CCK8, EdU, and colony formation assays were performed to explore the proliferation capacity of CRC cells. Transwell invasion and migration assays, along with the wound healing assay, were used to explore the invasive and migratory abilities of CRC cells. Subcutaneous Xenograft Assay was utilized to evaluate the tumorigenic capacity of CRC cells in vivo.

**Results:**

SULF1 was highly expressed in CRC samples and cell lines. The knockdown of SULF1 inhibited the proliferation, invasion, and migration of CRC and increased the rate of cell apoptosis. Meanwhile, we demonstrated that SULF1 could negatively regulate ARSH through the FAK/PI3K/AKT/mTOR pathway.

**Conclusion:**

We demonstrated that SULF1 could promote CRC progression by regulating ARSH. The SULF1/ARSH/FAK/PI3K/AKT/mTOR signaling pathway represents a promising target for the treatment of this patient population.

**Simple summary:**

Colorectal cancer (CRC) has the third highest incidence and second mortality rate of malignant tumors globally. Sulfatase 1 (SULF1) belongs to the sulfatase family, The function of SULF1 in CRC remains elusive. Our study demonstrated that the knockdown of SULF1 could inhibit the proliferation, invasion, and migration of CRC. Meanwhile, our findings indicated that SULF1 could interact with Arylsulfatase Family Member H (ARSH) to regulate the proliferation, invasion, and migration of CRC via the FAK/PI3K/AKT/mTOR signaling pathway. Taken together, our findings suggest that SULF1 might be a new therapeutic target in CRC.

**Supplementary Information:**

The online version contains supplementary material available at 10.1186/s12935-024-03383-5.

## Introduction

Colorectal cancer (CRC) is the third most common cancer globally, and its mortality rate ranks second worldwide [1]. Despite the abundance of treatment options for CRC (radical resection, radiotherapy, and chemotherapy), the 5-year survival rate of CRC patients remains dismally low [2, 3]. The progression of CRC and its sensitivity to chemotherapy and immunotherapy involve multiple significant signaling pathways in vivo, which is an extremely complex biological process [4]. Consequently, it is urgent to explore the potential mechanisms of CRC progression, which can provide better guidance for the treatment of this patient population.

Sulfatase 1 (SULF1) belongs to the sulfatase family, which plays its biological function by specifically removing the sulfate group of the heparan sulfate proteoglycan core protein (HSPG) 6-O on the cell surface [5]. HSPG is present on the cell surface and extracellular matrix of most animals, which can bind and regulate more than 400 bioactive proteins (including cytokines, chemokines, and growth factors) and protect them from protease hydrolysis [6, 7]. In addition, the side chain of HSPG heparan sulfate (HS) itself is the ligand of certain receptors and is involved in the activation of cell signaling pathways [7]. Consequently, SULF1 can regulate the ability of HSPG to bind a variety of protein ligands by regulating the conformation of HSPG, thereby regulating the progression of tumors [8]. However, the role of SULF1 in CRC remains unclear. Arylsulfatase Family Member H (ARSH) is now understood to be involved in the biosynthesis of hormones, regulation of cell signaling, and degradation of macromolecules [9]. However, the role of ARSH in human tumors, particularly colon cancer, remains largely unexplored.

Focal adhesion kinase (FAK) participates in various fundamental processes, encompassing proliferation, invasion, and metastasis in multiple tumors [10]. Previous studies have demonstrated that aberrant phosphorylation of FAK can promote the progression of CRC [11–13]. Protein kinase B (AKT), phosphatidylinositol-3-kinase (PI3K), and mechanistic target of rapamycin kinase (mTOR) have been documented as the downstream genes of FAK [14–17]. Studies have shown that SULF1 can activate the AKT pathway in hepatocellular carcinoma [18]; interestingly, a recent study also revealed that SULF1 was capable of activating the PI3K/AKT and the PI3K/AKT pathway in cervical cancer [19], promoting tumor development. However, its role in CRC through this pathway remains unknown. Therefore, our study aimed to investigate whether SULF1 could regulate tumor proliferation, invasion, and metastasis in CRC via the FAK/PI3K/AKT/mTOR axis.

In this study, we demonstrated that SULF1 was highly expressed through a combination of microarray data and in vitro experiments. Meanwhile, SULF1 inhibition significantly suppressed CRC cell proliferation, migration, and metastasis while concurrently upregulating the expression of ARSH and p-FAK activity. In conclusion, our studies indicated that SULF1 is vital to the progression of CRC and that SULF1 might be a new target for clinical targeted therapy of CRC.

## Materials and methods

### Acquisition of microarray data

In this study, one microarray dataset was downloaded from The Cancer Genome Atlas (TCGA, https://portal.gdc.cancer.gov/) database. Five microarray datasets were acquired from Gene Expression Omnibus (GEO, http://www.ncbi.nlm.nih.gov/geo) database, containing GSE41328 [20], GSE44076 [21], GSE44861 [22], GSE136735 [23], and GSE17536 [24]. In our study, data from all databases was accessed on December 1, 2023. The cohorts included: TCGA (26 normal colorectal specimens and 270 CRC specimens), GSE41328 (10 CRC specimens and 10 adjacent normal colorectal specimens), GSE136735 (6 CRC specimens and 6 adjacent normal colorectal specimens), GSE44076 (98 CRC specimens and 98 adjacent normal colorectal specimens), GSE44861 (55 CRC specimens and 55 adjacent normal colorectal specimens), and GSE17536 (177 CRC specimens). The microarray data from GSE41328 and GSE17536 were based on the GPL570 platform (HG-U133_Plus_2) Affymetrix Human Genome U133 Plus 2.0 Array. The microarray data from GSE44076 was based on the GPL13667 platform (HG-U219) Affymetrix Human Genome U219 Array. The microarray data from GSE44861 was based on the GPL3921 platform (HT_HG-U133A) Affymetrix HT Human Genome U133A Array. The microarray data from GSE136735 were based on the GPL16699 platform Agilent-039494 SurePrint G3 Human GE v2 8 × 60 K Microarray 039381 (Feature Number version). In addition, TCGA and GSE17536 cohorts included 270 and 177 CRC patients’ clinical data respectively. We further merged these cohorts into a single dataset called the Entire cohort.

### Identification of *SULF1*

Firstly, we used the “RMA” and “Affy” packages in R studio to identify differentially expressed genes (DEGs) between CRC and normal colorectal specimens by analyzing microarrays from GSE41328, GSE44076, GSE44861 and GSE136735 based on the screening criteria |logFC|>1 and adjusted *p* < 0.05. A Venn plot was generated to screen out the overlapping DEGs among datasets GSE41328, GSE44076, GSE44861 and GSE136735. Subsequently, we constructed a protein–protein interaction (PPI) network of these overlapping DEGs using Search Tool for the Retrieval of Interacting Genes (String, http://string-db.org; Version:11.0) online database [25]. The CytoHubba plug-in in Cytoscape software was used to identify the top 10 hub genes of the PPI network. *SULF1* was one of the top 10 hub genes. Lastly, we explored the expression levels, overall survival (OS) and disease-free survival (DFS) of 10 hub genes in CRC patients according to gene expression profiling interactive analysis (GEPIA, http://gepia.cancer-pku.cn/) online database. Notably, *SULF1* exhibited statistically significant associations with both OS and DFS. Hence, we chose *SULF1* for our subsequent study.

### GO (Gene Ontology) and KEGG (Kyoto Encyclopedia of genes and genomes) enrichment analyses

We used cBioPortal (https://www.cbioportal.org/) [26] online database to identify 361 co-expression genes of SULF1 (|Spearman| > 0.9; Supplementary Table 6). Based on these co-expression genes, “ggplot2”, “enrichplot”, “org.Hs.eg.db”, and “clusterProfiler” packages were used to perform GO and KEGG enrichment analyses.

### Patient specimens and cell culture

All CRC tissues and adjacent cancer tissues were collected from the Second Affiliated Hospital of Nanchang University (Nanchang, China) between September 2018 and September 2023. All of these patients did not receive any radiotherapy or chemotherapy before surgery. The collected tissues were then stored in 4% paraformaldehyde or at -80℃.

All cell lines were purchased from the Cell Bank of the Chinese Academy of Sciences (Shanghai, China). HCT116 was cultured in RPMI-1640 medium supplemented with penicillin G (100 mg/mL), streptomycin (100 mg/mL), and 10% fetal bovine serum (FBS; Gibco; USA). NCM460, SW620, SW480, HT29, and DLD1 were cultured in DMEM medium with penicillin G (100 mg/mL), streptomycin (100 mg/mL), and 10% fetal bovine serum (FBS; Gibco; USA). All cells were incubated in a 37 °C incubator with 5% CO_2_.

### Cell transfection

Lentiviruses for SULF1 silencing (sh-SULF1#1, sh-SULF1#2, and sh-SULF1#3) and the silencing control (NC) were purchased from HANBIO (Shanghai, China). ARSH plasmid (ARSH) and ARSH siRNA (si-ARSH) were purchased from Genechem (Shanghai, China). Lentiviral transduction was performed according to the manufacturer’s instructions. For plasmid and siRNA transfection, we used Lipofectamine 3000 Transfection Reagent (Invitrogen, Waltham, Massachusetts, USA).

### Quantitative real-time PCR (qRT-PCR)

Total tissue and cell RNA were extracted using the Trizol method, which were reverse transcribed into cDNA (TAKARA, RR047A). The cDNA was used for real-time quantitative PCR (TAKARA, RR420A). The 2^− ρρCt^ method was used for data analysis.

The primer sequences used are presented in Supplementary Table 9.

### Cell proliferation assay

EdU, CCK8 and colony formation assays were performed to testify proliferation capacity of HCT116 and SW480 cells. For the EdU assay, cells were seeded in 96-well plates with 2 × 10^4^ cells per well. After the cells were incubated at 37 ℃ for 8 h, EDU incubation, fixation, and staining were performed according to the instructions of the YF®594 Click-iT EDU staining kit (UE, Shanghai, China). Photographs were taken under a fluorescence microscope finally. For CCK8 assay, cells (5000 cells/well) were inoculated in a 96-well plate. The medium containing 10% CCK-8 (Biosharp, Beijing, China) was added to each well at the specified time (6 h, 24 h, 48 h, 96 h). After incubation at 37℃ for 2 h, the absorbance of each well was detected at 450 nm, and the data were analyzed to evaluate the cell proliferation ability. For colony formation assay, cells (1000 cells/well) were seeded into 6-well plates. After incubation for two weeks, the cells were fixed with 4% paraformaldehyde, stained with crystal violet, and photographed to calculate the number of spherical cells to evaluate the proliferation ability of the cells.

### Transwell assay

We performed Transwell invasion and migration assays to explore cell invasion and migration ability respectively. For the invasion assay, each chamber was pre-coated with Matrigel diluted 1:8 in medium. For both invasion and migration assays, 3 × 10^4^ HCT116 or SW480 cells were seeded in 200 µL serum-free medium into each upper chamber while 600 µl complete medium with 20% fetal bovine serum were filled with each lower chamber. After 48–72 h of incubation, HCT116 or SW480 cells that invaded the lower chamber were fixed with 4% paraformaldehyde and stained with crystal violet.

### Wound healing assay

Wound healing assay was performed to explore the migration capacity of HCT116 and SW480 cells. 6 × 10^4^ HCT116 or SW480 cells per well were seeded into six-well plate.

We performed the wound healing assay using a 200µL sterile pipette when cell monolayers were adherent. The complete medium was then replaced with serum-free medium to minimize cell proliferation and promote migration. Finally, wound closure was monitored by photographing the wells at 0 and 24 h.

### Cell apoptosis and cell cycle

For cell apoptosis, the cells (10^4^ cells /mL) were seeded in a 6-well plate. After cell growth reached 60-70%, the cells were collected, washed with pre-cooled phosphate-buffered saline (PBS) without Ca^2+^ and Mg^2+^, and then centrifuged. Annexin V-FITC and PI (UE, Shanghai, China) were then added and incubated for 15 min in the dark. Finally, 400µL buffer was added for re-suspension and then detected by flow cytometry. For cell cycle, cells were collected, centrifuged, washed with pre-cooled PBS, fixed with pre-cooled 70% ethanol, and placed in a -20℃ refrigerator overnight. On the next day, the fixed cells were washed with pre-cooled PBS, and PI (UE, Shanghai, China) were added after washing. Cell cycle detection was performed by flow cytometry.

### Western blotting assay

Total protein lysates were extracted from HCT116 and SW480 cells using radioimmunoprecipitation assay buffer (Beyotime Institute of Biotechnology, Shanghai, China). The lysates were separated by SDS-PAGE and blotted onto PVDF membranes (Bio-Rad). Then, we blocked the protein-free sites on PVDF membranes with 5% skim milk. Subsequently, the PVDF membranes were incubated with antibodies against SULF1 (1:1000, Affinity, DF13592), ARSH (1:1000, Affinity, DF9228), FAK antibody (1:1000, ZENBIO, 381,143), p-FAK antibody (1:1000, ZENBIO, R24276), PI3K antibody (1:1000, ZENBIO, 251,221), p-PI3K antibody (1:1000, ZENBIO, 310,164), AKT antibody (1:1000, ZENBIO, 342,529), p-AKT antibody (1:1000, ZENBIO, 310,021), mTOR antibody (1:5000, Proteintech, 66888-1-Ig), p-mTOR antibody (1:2000, Proteintech, 67778-1-Ig), BAX antibody (1:2000, Proteintech, 50599-2-Ig), cleaved caspase-3 antibody (1:500, Abcam, ab2302), BCL2 antibody (1:5000, Proteintech, 68103-1-Ig), CDK4 (1:1000, Affinity, DF6102), CDK6(1:1000, Affinity, DF6448), CyclinD1(1:1000, Affinity, AF0931), E-cadherin(1:1000, Affinity, AF0131), N-cadherin(1:1000, Affinity, AF5239), Snail(1:1000, Affinity, AF6032), and GAPDH (1:50000, Proteintech, 60004-1-Ig) overnight at 4℃. Then, the PVDF membranes were probed with secondary HRP Linked Secondary Antibodies (Sangon Biotech, Shanghai.). Finally, we used imaging system (DenvilleScientific Inc., Holliston, MA, USA) to visualize immunoblots. Details of all primary antibodies are provided in Supplementary Table 10.

### Immunoprecipitation (IP)

Cells were lysed on ice for 30 min with pre-cooled radio immunoprecipitation assay (RIPA) buffer containing phenylmethanesulfonyl fluoride (PMSF) (100:1 ratio). The lysate was then centrifuged at 10,000 x g for 10 min. The supernatant was added with 2 µg primary antibody and incubated in a shaker for 1 h, then 40 µL protein A/G PLUS-Agarose (Santa Cruz, sc-2003) was added and incubated in a shaker at 4℃ for 12 h. The precipitation was collected after centrifugation at 2500 rpm for 5 min. The pellet was washed four times with pre-cooled 10% RIPA buffer. Finally, the precipitate was dissolved in 40 µL of 2× electrophoresis sample buffer, boiled for 10 min, and then western blotting was performed.

### Subcutaneous xenograft assay

Ten 4-week-old BALB/c female nude mice, weighing 18–21 g, were purchased from Keris Biotechnology Company, Nanjing, and raised in a specific-pathogen free (SPF) environment.

Ten female BALB/c nude mice were randomly divided into 2 groups: Non-targeting control (NC) group and sh-SULF1#1 group. Cell suspensions (1 × 10^7^) of HCT116 cells stably transfected with either NC or sh-SULF1#1 were added to 200 µL of PBS. To ensure high biological activity during the experiment, these cells were in a logarithmic growth phase. The skin on the right flank of the nude mice was disinfected with alcohol, and 100 µL of each cell suspension was then injected into the area using a 1 mL syringe. The width and length of the tumor were measured at 5-day intervals, and the tumor was calculated by the formula (V): V = 0.52× length × width^2^. After 40 days of inoculation, the nude mice were euthanized by means of carbon dioxide (CO_2_) inhalation. The euthanasia chamber was utilized to expose the nude mice to CO_2_ at a flow rate equivalent to 20% of the replacement volume per minute. Once complete immobility, respiratory arrest, and pupil dilation were observed in the nude mice, the administration of CO_2_ was terminated. Following a 3-minute observation period to confirm death, the nude mice could be removed from the euthanasia chamber. Subsequently, tumor tissue was extracted for photographic analysis. All animal assays were approved by the Laboratory Animal Science Center of Nanchang University.

### Immunohistochemistry (IHC)

The tumor tissue was embedded in paraffin blocks and cut into Sect. 4 μm thick. Tissue sections were incubated with primary and then enzyme-labeled secondary antibodies. Positive staining was visualized using 3,3’-diaminobenzidine (DAB) as the chromogen. IHC staining results from patient or mouse tissues were then quantified using the positive staining cell count method. Protein expression was categorized into a four-tier score (0, 1, 2, or 3) based on the percentage of positively stained cells: 0–5%, 6–25%, 26–50%, and 51–100%. Scores of 0 and 1 were classified as low expression, while 2 and 3 were classified as high expression.

### Statistical analysis

GraphPad Prism (version: 8.0.1) and R studio (version: 4.0.3) were used for data analyses Student’s t-test was employed to compare the means between two groups. One-way ANOVA test was employed to assess the means among multiple groups. Kaplan-Meier survival curves were generated and compared using the Log-rank test. All experiments were independently repeated three times. Statistical significance was considered at *p* < 0.05.

## Results

### Identification of *SULF1*

**Fig. 1:**
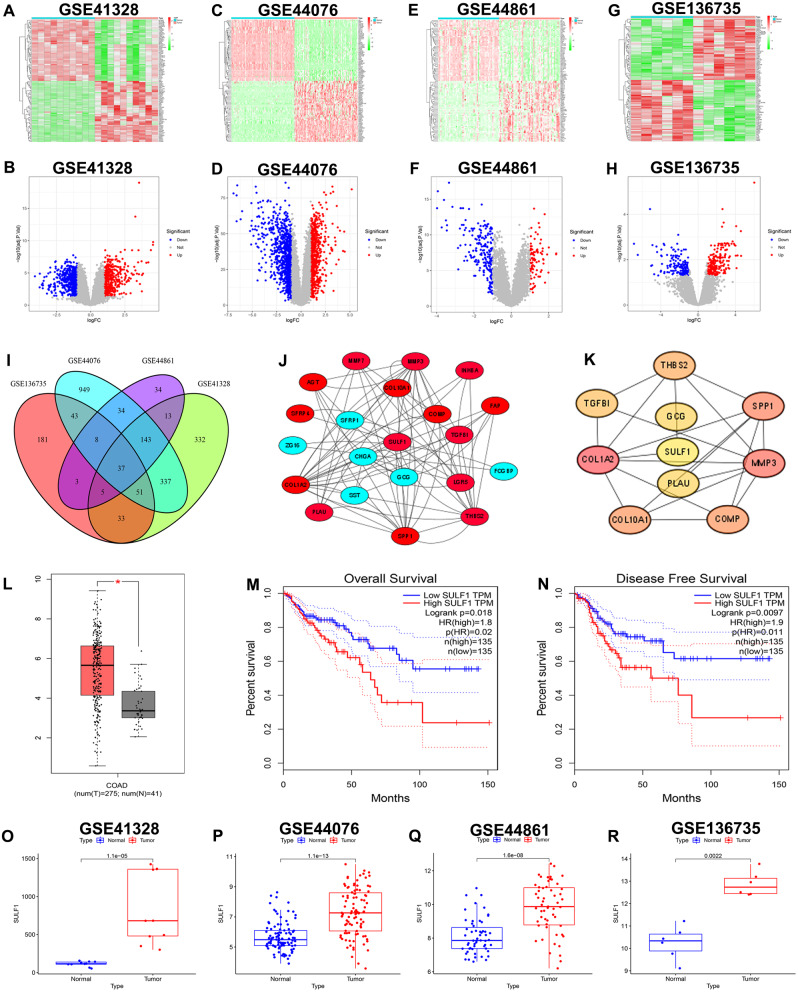
Identification of *SULF1*. **(A)**. Heatmap of DEGs in GSE41328. **(B)**. Volcano plot of DEGs in GSE41328. **(C)**. Heatmap of DEGs in GSE44076. **(D)**. Volcano plot of DEGs in GSE44076. **(E)**. Heatmap of DEGs in GSE44861. **(F)**. Volcano plot of DEGs in GSE44861. **(G)**. Heatmap of DEGs in GSE136735. **(H)**. Volcano plot of DEGs in GSE136735. **(I)**. The Venn diagram showed 37 overlapping DEGs. **(J)**. PPI network generated based on the 37 overlapping DEGs. Up-regulated and down-regulated genes are marked in red and blue respectively. **(K)**. Top 10 hub genes in the PPI network, which were calculated by method MCC. **(L)**. Box plot showing differential mRNA expression of *SULF1* between normal colorectal tissue and CRC tissue based on the GEPIA database. **(M)**. The Kaplan-Meier curve of OS of *SULF1* from GEPIA database. **(N)**. The Kaplan-Meier curve of OS of *SULF1* from GEPIA database. **(O)**. Differential mRNA expression of *SULF1* between normal colorectal tissue and CRC tissue in GSE41328. **(P)**. Differential mRNA expression of *SULF1* between normal colorectal tissue and CRC tissue in GSE44076. **(Q)**. Differential mRNA expression of *SULF1* between normal colorectal tissue and CRC tissue in GSE44861. **(R)**. Differential mRNA expression of *SULF1* between normal colorectal tissue and CRC tissue in GSE136735. All experiments were repeated three times and the data were represented as mean ± SD. *p < 0.05, ** *p* < 0.01, ***p < 0.001, ****p < 0.0001

To identify a key gene correlated with prognosis in CRC we initially conducted a comprehensive bioinformatics analysis. Firstly, according to the cut-off threshold of |logFC|>1 and adjusted *p* < 0.05, we identified 951, 1602, 277, 361 DEGs by analyzing GSE41328 (Fig. 1A, B; Supplementary Table 1), GSE44076(Fig. 1C, D; Supplementary Table 2), GSE44861(Fig. 1E, F; Supplementary Table 3) and GSE17536(Fig. 1G, H; Supplementary Table 4) microarray data respectively. Then, we identified 37 overlapping DEGs across GSE41328, GSE44076, GSE44861, and GSE136735 datasets, and the results were visualized in a Venn diagram (Fig. 1I). Subsequently, the STRING online database was used for PPI network analysis of the overlapping DEGs, and Cytoscape software was used for its visualization (Fig. 1J). Next, according to the MCC method in CytoHubba plug-in of Cytoscape software, top 10 hub genes (*SULF1*, *GCG*, *PLAU*, *THBS2*, *TGFB1*, *COL1A2*, *COL10A1*, *COMP*, *MMP3*, and *SPP1*) in PPI network were identified (Fig. 1K; Supplementary Table 5). Following, we used GEPIA online database for prediction of mRNA expression level (Fig. 1L, Supplementary Fig. 1), OS (Fig. 1M, Supplementary Fig. 2), and DFS (Fig. 1N, Supplementary Fig. 3) of 10 hub genes. Interestingly, only *SULF1* exhibited statistically significant associations with mRNA expression (Fig. 1L), OS (Fig. 1M), and DFS (Fig. 1N). In addition, *SULF1* expression was significantly higher in CRC specimens compared to adjacent normal colorectal specimens in GSE41328 (Fig. 1O), GSE44076 (Fig. 1P), GSE44861 (Fig. 1Q), and GSE136735 (Fig. 1R) cohorts. Therefore, considering the significant differential expression of *SULF1* across multiple databases and its strong correlation with prognosis in CRC, we ultimately chose *SULF1* as the key gene for our subsequent investigation.

### SULF1 is upregulated in CRC

**Fig. 2:**
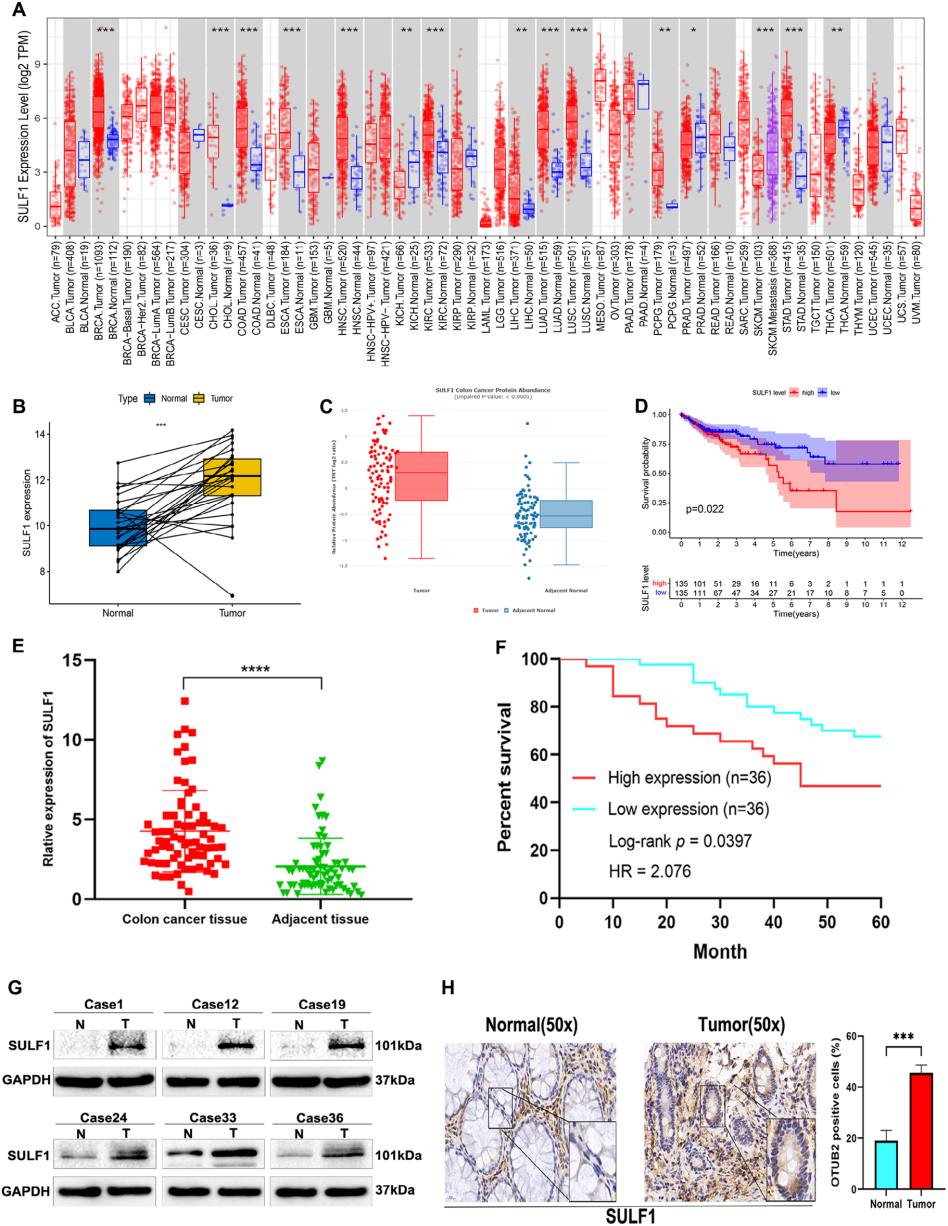
SULF1 is upregulated in CRC samples. **(A)**. Differential expression analysis of *SULF1* in multiple tumors from TIMER database. The red dots represent tumor samples, the blue dots represent normal sample. **(B)**. Analysis of differential mRNA expression in paired samples of CRC from TCGA cohort. **(C)**. Analysis of differential protein expression in CRC from CPTAC database. **(D)**. Kaplan-Meier curve of OS of *SULF1* from TCGA cohort. **(E)**. Expression of *SULF1* in 72 pairs of tumorous and para-carcinoma tissue from the Second Affiliated Hospital of Nanchang University. **(F)**. Kaplan-Meier curve of OS of *SULF1* was performed using 72 CRC samples from the Second Affiliated Hospital of Nanchang University. **(G)**. Western blotting showed the protein expression of SULF1 in tumorous and para-carcinoma tissue. **(H)**. Immunohistochemistry (IHC) showed the protein expression of SULF1 in tumorous and para-carcinoma tissue. All experiments were repeated three times and the data were represented as mean ± SD. *p < 0.05, ** *p* < 0.01, ***p < 0.001, ****p < 0.0001

Our comprehensive screening across multiple databases identified *SULF1* as a key gene in CRC. To investigate its functional role, we examined *SULF1* mRNA and protein expression levels and their correlation with patient prognosis using a combination of bioinformatics analysis and our own experimental data. The results derived from bioinformatics analyses were as follows:

First, the mRNA expression of *SULF1* was examined across various cancer types, revealing significant variations in its expression levels, including CRC (Fig. 2A). This finding underscored the pivotal role of *SULF1* in the initiation and progression of human tumors. Besides, we explored the differential mRNA expression of *SULF1* in 26 pairs of CRC samples from TCGA database, which also showed that *SULF1* was highly expressed in CRC samples (Fig. 2B). Subsequently, the Clinical Proteomic Tumor Analysis Consortium (CPTAC) database was utilized to investigate the protein expression level of SULF1 in CRC. The results revealed higher protein expression of SULF1 in CRC tissues compared to normal colorectal tissues (Fig. 2C). Finally, to investigate the correlation between *SULF1* expression and prognosis in patients with CRC, we divided 270 CRC patients from TCGA cohort into high and low expression groups according to the median expression of *SULF1*. The Kaplan-Meier curve revealed that the OS of the high expression group was significantly lower than the low expression group (*p* = 0.022), which demonstrated that CRC patients with higher expression level of *SULF1* had a worse prognosis (Fig. 2D). Therefore, our bioinformatics analyses demonstrated a significant upregulation of SULF1 in CRC and its close association with the OS of this patient population.

Subsequently, we conducted analyses on a total of 72 pairs of CRC samples that were collected by our team from the Second Affiliated Hospital of Nanchang University in order to further validate the results obtained through bioinformatics analyses. The results were as follows:

First, the qRT-PCR assay revealed that the mRNA expression level of *SULF1* was significantly elevated in CRC tissues compared to adjacent tissues (Fig. 2E**)**. Next, Western blotting and IHC assays showed significantly elevated protein expression of SULF1 in CRC tissues compared to adjacent tissues. (Fig. 2G, H**)**. Finally, to investigate the correlation between *SULF1* expression and prognosis in CRC patients, the patients (*n* = 72) were categorized into the high-expression and low-expression groups based on the median value of *SULF1* expression. Table 1 summarizes the association between *SULF1* expression and clinicopathological characteristics. Notably, the data revealed a significant correlation between high *SULF1* expression and both tumor diameter and TNM stage. Besides, Kaplan-Meier analysis utilizing patient follow-up data also indicated that high *SULF1* expression was associated with unfavorable patient prognosis (Fig. 2F). Hence, analyses of our own samples revealed significant upregulation of SULF1 in CRC and its close association with the prognosis of this patient population.

In conclusion, our comprehensive analyses integrating bioinformatic data and experimental validation in our own samples demonstrated significant upregulation of SULF1 in CRC tissues. Furthermore, these analyses revealed a strong correlation between SULF1 expression and clinical outcomes in CRC patients.

**Table 1:** The association between *SULF1* expression and clinicopathological characteristics

Characteristics	Low expression of SULF1	High expression of SULF1	*p* value
n	32	32	
Age, n (%)			0.7928
≤ 50	12(18.75%)	10 (15.63%)	
> 50	20(31.25%)	22(34.38%)	
Gender, n (%)			0.6029
Male	22 (34.38%)	19 (29.69%)	
Female	10 (15.63%)	13 (20.31%)	
Diameter of tumor(cm), n (%)			**0.0118**
≤ 5	22 (34.38%)	11 (17.19%)	
> 5	10 (15.63%)	21 (32.81%)	
TNM stage, n (%)			**0.0420**
I/II	23 (35.94%)	14 (21.88%)	
III/IV	9 (14.06%)	18 (28.13%)	
Lymphatic metastasis, n (%)			0.7928
Negative	22 (34.38%)	20 (31.25%)	
Positive	10 (15.63%)	12 (18.75%)	
Distant metastasis, n (%)			0.3020
Negative	29 (45.31%)	25 (39.06%)	
Positive	3 (4.69%)	7 (10.94%)	

### Knocking down SULF1 inhibits CRC cell proliferation, migration, and invasion in vitro

**Fig. 3:**
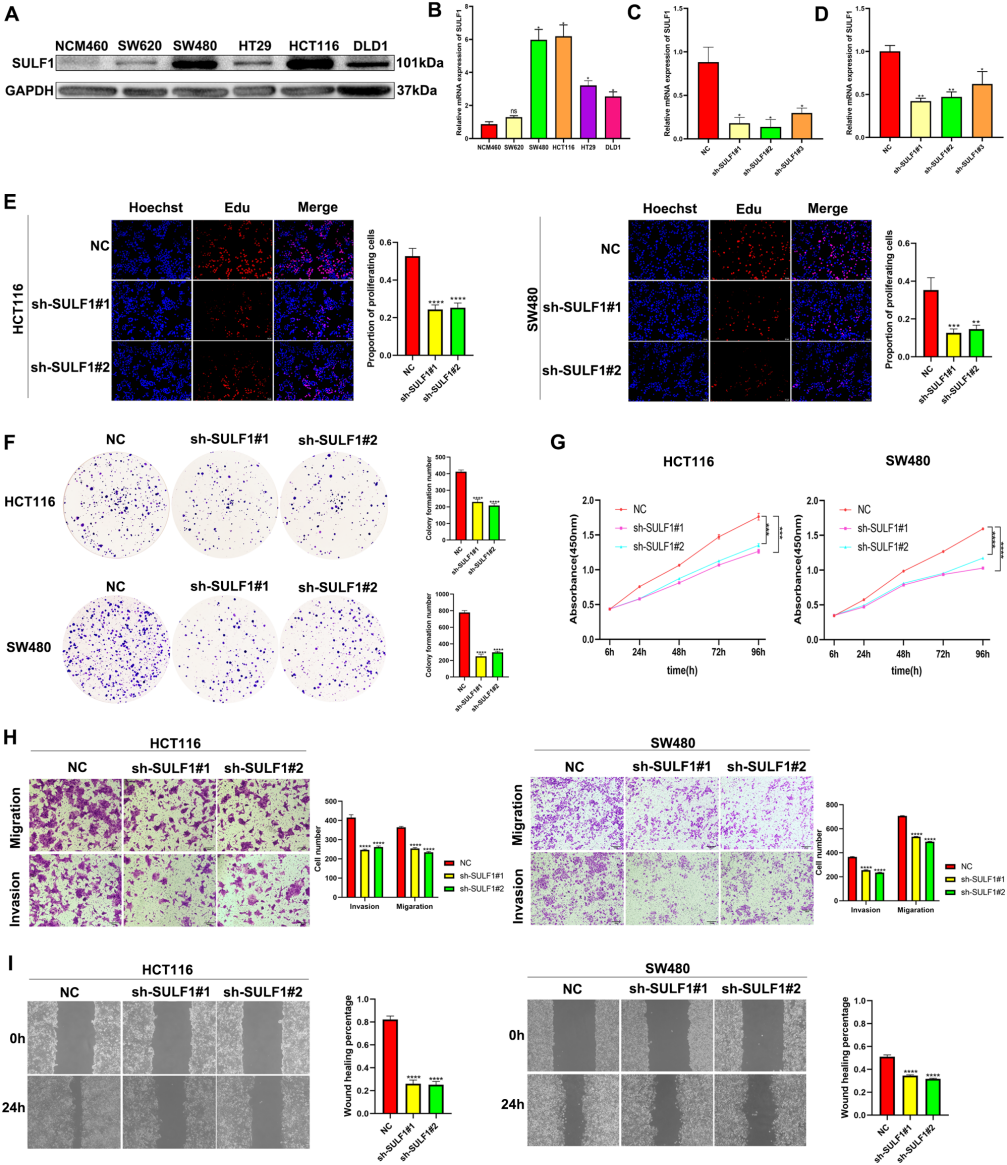
SULF1 knockdown inhibits CRC cell proliferation, migration, and invasion in vitro. **(A)** Protein expression of SULF1 in normal cell line and colon cancer cell lines. **(B)** mRNA expression of *SULF1* in normal colon cell line and colon cancer cell lines. **(C)** The transfection efficiency of siRNA was verified by qRT-PCR in HCT116. **(D)** The transfection efficiency of siRNA was verified by qRT-PCR in SW480. The EdU assay**(E)**, colony formation assay**(F)**, and CCK8 assay**(G)** were performed to verify the proliferative ability of HCT116 and SW480. **(H)** Transwell invasion and migration assays were performed to verify the invasion and migration ability of HCT116and SW480. **(I)** Wounding healing assay was performed to verify the migration ability of HCT116 and SW480. All experiments were repeated three times and the data were represented as mean ± SD. *p < 0.05, ** *p* < 0.01, ***p < 0.001, ****p < 0.0001

Our previous results demonstrated significant upregulation of SULF1 in CRC and its close association with the prognosis of CRC patients. It is well-established that changes in patient prognosis are often closely linked to alterations in tumor cell proliferation, invasion, and migration capabilities [27]. Hence, we investigated the potential impact of SULF1 on CRC cell proliferation, invasion, and migration capabilities. Firstly, we explored the protein and mRNA expression of *SULF1* in normal colon cell line (NCM460) and colon cancer cell lines (SW620, SW480, HT29, HCT116, and DLD1) by western blotting (Fig. 3A) and qRT-PCR (Fig. 3B), which revealed that the protein and mRNA expression were higher in all colon cancer cell lines compared with normal colon cell line. Given that HCT116 and SW480 cell lines had the highest protein and mRNA expression levels in all five CRC cell lines, they were selected for our subsequent studies. Then, the HCT116 and SW480 cell lines were both transduced with lentivirus encoding short interfering RNAs for *SULF1* silencing. The efficiency of *SULF1* knockdown was evaluated by qRT-PCR. The qRT-PCR analysis showed better knockdown efficiency of sh-SULF1#1 and sh-SULF1#2 in HCT116 cells than sh-SULF1#3 (Fig. 3C). The qRT-PCR analysis of SW480 cells (Fig. 3D) yielded results consistent with those observed in HCT116 cells. Hence, sh-SULF1#1 and sh-SULF1#2 were chosen for subsequent experiments.

Subsequently, we employed EdU, colony formation, and CCK-8 assays to validate the impact of SULF1 on the proliferative capacity of CRC cells. The results demonstrated that knocking down SULF1 significantly inhibited proliferation of HCT116 and SW480 cells (Figure E-G). Then, the Transwell migration assay and wound-healing assay were employed to validate the impact of SULF1 on the migration capacity of CRC cells. The results revealed that knockdown of SULF1 significantly attenuated migration of HCT116 and SW480 cells (Figure H, I). Finally, Transwell invasion assay was employed to validate the impact of SULF1 on the invasion capacity of CRC cells. The results demonstrated that knockdown of SULF1 decreased the abundance of HCT116 and SW480 cells that invaded through the matrigel-coated chamber (Figure H). Overall, these results revealed that knockdown of SULF1 inhibited the proliferation, migration, and invasion of HCT116 and SW480 cells.

### Effects of SULF1 on CRC cell cycle and apoptosis

**Fig. 4:**
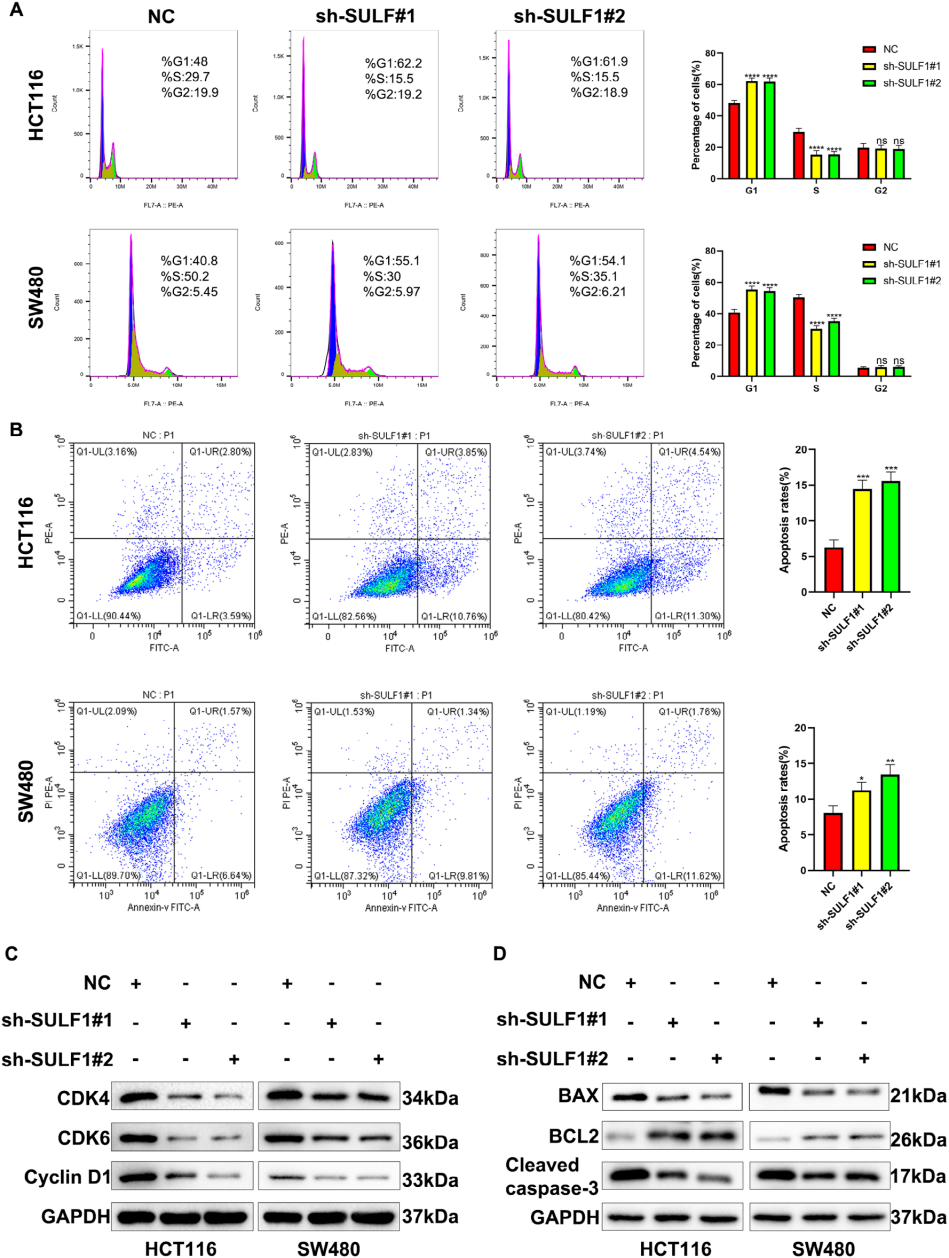
SULF1 knockdown facilitates cell cycle arrest and induces the apoptosis of CRC cells. **(A)** SULF1 knockdown can increase the proportion of G2 phase cells and decrease the proportion of S phase cells in HCT116 and SW480. **(B)** The apoptosis rate of sh-SULF1#1 and sh-SULF1#2 groups were higher than that of NC group. **(C)** Western blotting showed that SULF1 knockdown could decrease the protein expression of cell cycle markers, CDK4, CDK6, and CyclinD1. **(D)** Western blotting showed that SULF1 knockdown could decrease the protein expression of apoptosis markers, Bax and Cleaved caspase-3, but increase Bcl-2. All experiments were repeated three times and the data were represented as mean ± SD. *p < 0.05, ** *p* < 0.01, ***p < 0.001, ****p < 0.0001

Our previous work established the influence of SULF1 on CRC cell proliferation. Given the well-documented link between dysregulated cell cycle progression and tumor cell proliferation, with apoptosis acting as a counteracting force [28], we aimed to investigate the potential association between SULF1 and both cell cycle progression and apoptosis in CRC cell. Flow cytometry was used to explore the effect of SULF1 on the cell cycle and cell apoptosis. In both HCT116 and SW480 cells, compared with sh-SULF1#1 and sh-SULF1#2 groups, the proportion of S phase cells in NC group was significantly reduced, and the proportion of G1 phase cells was significantly increased (Fig. 4A). Meanwhile, the apoptotic HCT116 and SW480 cells significantly increased when SULF1 was knocked down (Fig. 4B). Western blotting analysis also revealed that cell cycle and apoptosis-related proteins, such as CDK4, CDK6, CyclinD1, Bax, and Cleaved caspase-3 were downregulated in sh-SULF1#1 and sh-SULF1#2 groups, while the protein expression level of Bcl-2 was significantly upregulated (Fig. 4C, D). In conclusion, these results demonstrated that knockdown of SULF1 could inhibit CRC progression by inhibiting G1/S phase progression and promoting apoptosis.

### Knockdown of SULF1 suppresses CRC EMT via the FAK/PI3K/AKT/mTOR pathway

**Fig. 5:**
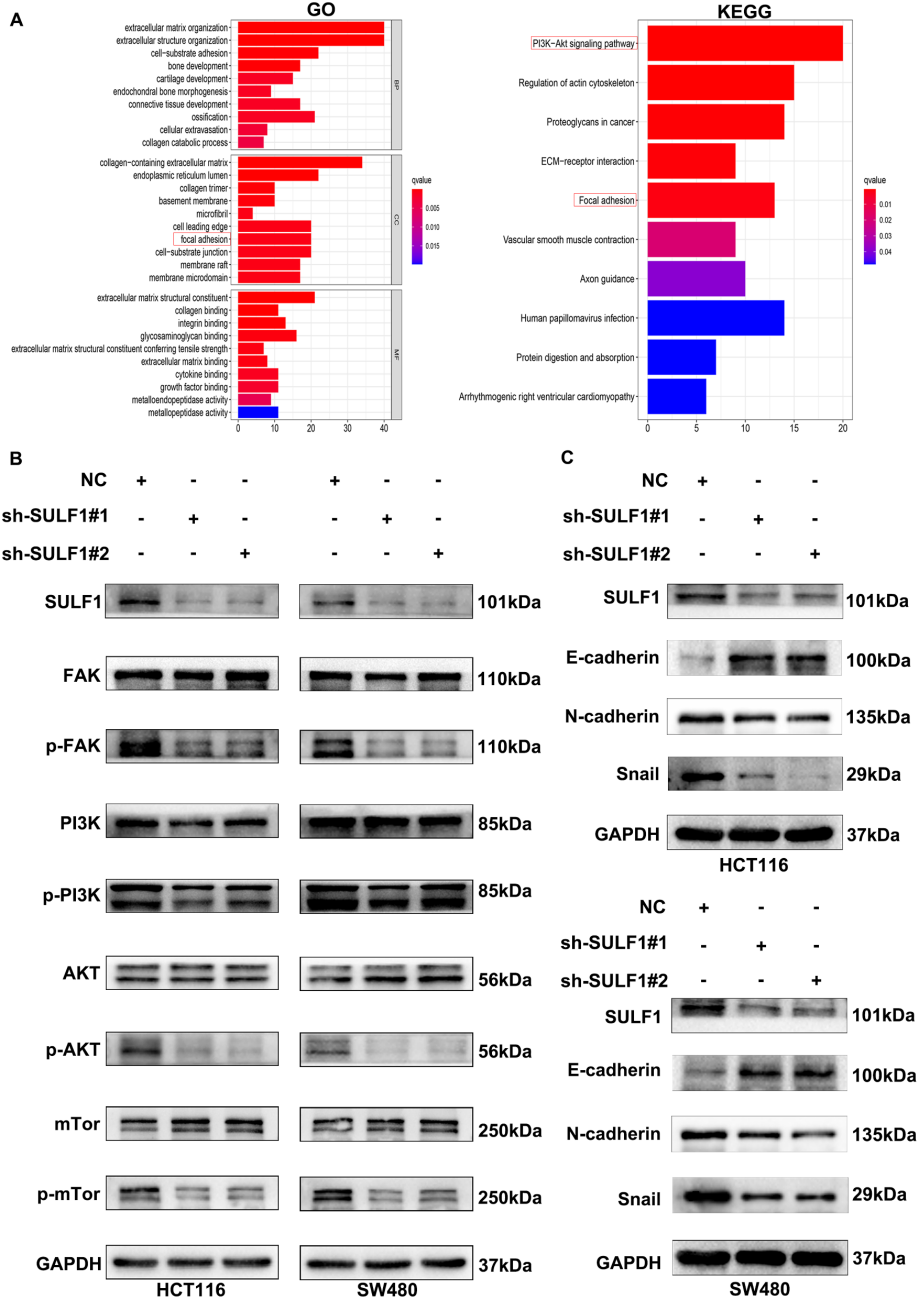
SULF1 knockdown inhibits EMT in CRC cells through the FAK/PI3K/AKT/mTor pathway. **(A)** GO and KEGG enrichment analyses showed that SULF1 was involved in CRC progression via the FAK/PI3K/AKT/mTor pathway. **(B)** Western blotting showed that SULF1 knockdown could decrease the protein expression of p-FAK, p-PI3K, p-AKT, and p-mTor. **(C)** Western blotting showed that SULF1 knockdown could decrease the protein expression of EMT markers, N-cadherin and Snail, but increase E-cadherin. All experiments were repeated three times and the data are represented as mean ± SD. *p < 0.05, ** *p* < 0.01, ***p < 0.001, ****p < 0.0001

To elucidate the biological processes underlying SULF1’s role in CRC progression, we performed Gene Ontology (GO) and Kyoto Encyclopedia of Genes and Genomes (KEGG) enrichment analyses. GO analysis revealed that focal adhesion was a potential mechanism by which SULF1 affects CRC (Fig. 5A; Supplementary Table 7). Consistently, KEGG analysis also showed that SULF1 could regulate both focal adhesion and PI3K-AKT signaling pathway (Fig. 5A; Supplementary Table 8). Hence, we speculated that SULF1 could regulate CRC progression through the FAK/PI3K/AKT/mTOR signaling pathway. Western blotting was utilized to validate this hypothesis. The results showed that the protein expression of p-FAK, p-PI3K, p-AKT, and p-mTOR was reduced following SULF1 knockdown (Fig. 5B). Since p-FAK induces the expression of EMT markers [29], we continued to explore whether SULF1 affects EMT in CRC. Western blotting revealed significant downregulation of the protein expression level of N-cadherin and Snail in sh-SULF1#1 and sh-SULF1#2 groups, while the protein expression level of E-cadherin was significantly upregulated (Fig. 5C). Overall, the above results substantiated that knockdown of SULF1 could inhibit EMT transition via the FAK/PI3K/AKT/mTOR Pathway during CRC progression.

### Knockdown of SULF1 inhibits CRC growth in vivo

**Fig. 6:**
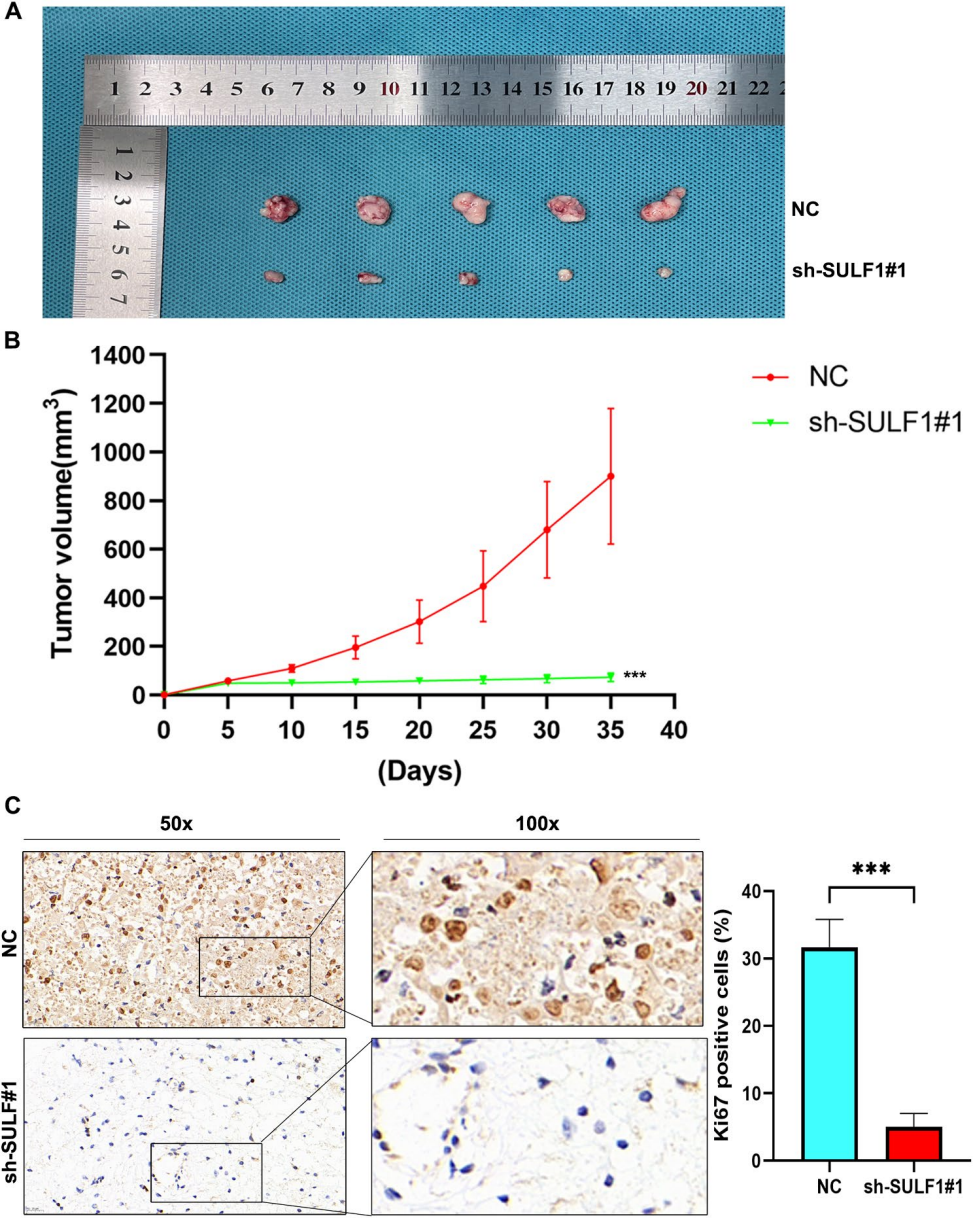
SULF1 knockdown inhibits CRC growth in vivo. **(A)** The tumor volume in the sh-SULF1#1 group were significantly smaller than that in the NC group. **(B)** The tumor volume was calculated at each specific time point and the results showed that tumor growth rate and volume in NC group were significantly higher than those in sh-SULF1#1 group. **(C)** IHC was performed to testify the protein expression of Ki67 in tumors. All experiments were repeated three times and the data are represented as mean ± SD. *p < 0.05, ** *p* < 0.01, ***p < 0.001, ****p < 0.0001

Subsequently, to further explore the effect of SULF1 in the growth of CRC in vivo, HCT116 cells from NC group and sh-SULF1#1 group were injected subcutaneously into the mice. The results showed that the tumor size in the sh-SULF1#1 group was significantly smaller than in the NC group (Fig. 6A). Besides, the tumor growth rate in the sh-SULF1#1 group was significantly lower than in the NC group (Fig. 6B). In addition, IHC showed that the protein expression of Ki67 was lower in the sh-SULF1#1 group compared to the NC group (Fig. 6C). Hence, these results indicated that knockdown of SULF1 could suppress CRC growth in vivo.

### SULF1 affects CRC proliferation, migration, and invasion by regulating ARSH in vitro

**Fig. 7:**
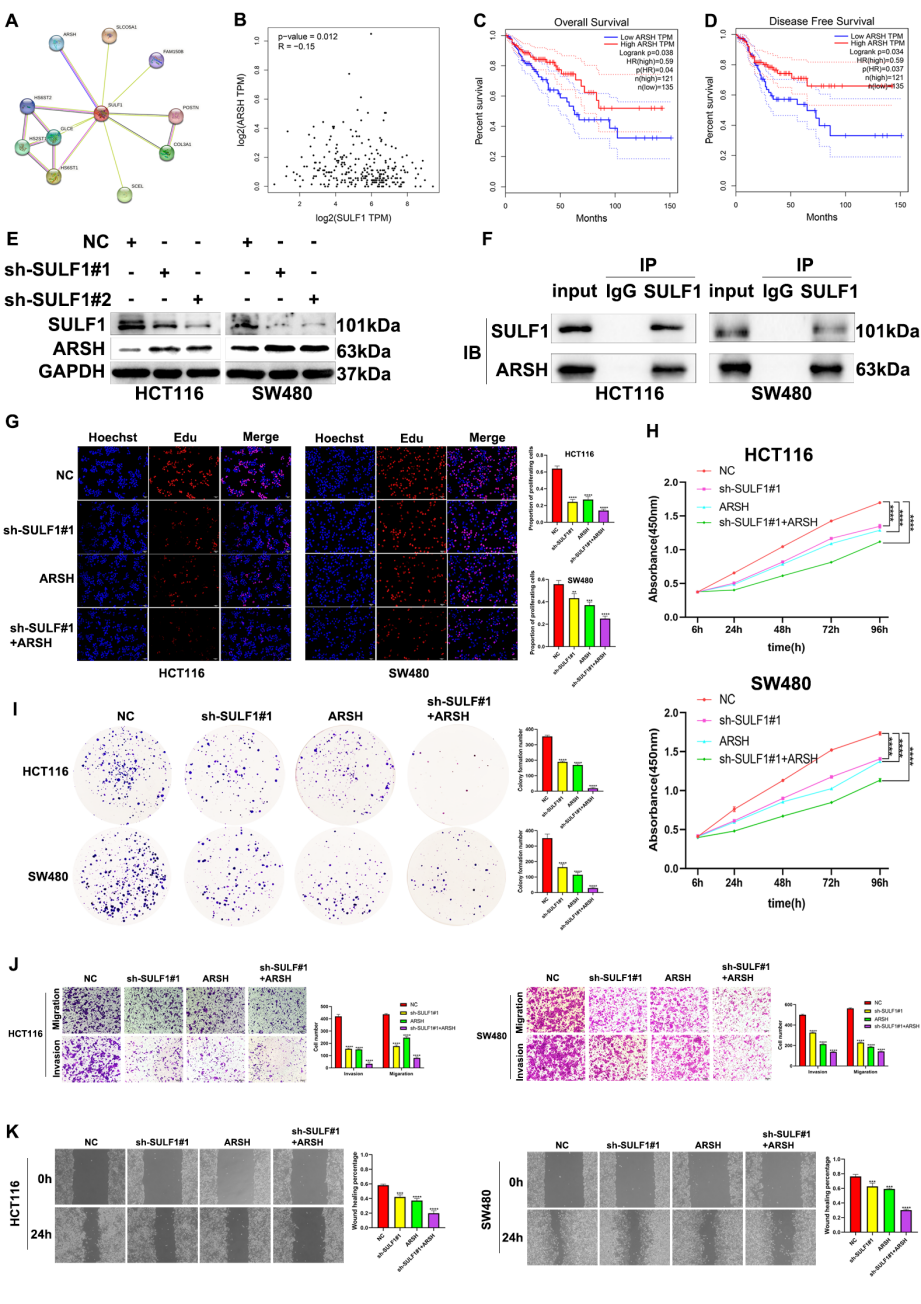
SULF1 influences proliferation, migration, and invasion of CRC cells by regulating ARSH. **(A)** STRING analysis showed that SULF1 interacted with ARSH. **(B)** SULF1 was significantly negatively correlated with ARSH in TCGA cohort from GEPIA database. **(C)** The Kaplan-Meier curve of OS of ARSH from GEPIA database. **(D)** The Kaplan-Meier curve of DFS of ARSH from GEPIA database. **(E)** Western blotting results showed that SULF1 was negatively correlated with ARSH protein expression. **(F)** Co-immunoprecipitation indicated the presence of direct or indirect binding between SULF1 and ARSH. **(G-I)** EdU assay, CCK-8 assay and colony formation assay were used to compare the proliferation ability of HCT116 and SW480 cells in different groups. **(J)** Transwell invasion and migration assays were performed to compare the invasion and migration ability of HCT116and SW480 in different groups. **(K)** Wounding healing assay was performed to compare the migration ability of HCT116 and SW480 in different groups. All experiments were repeated three times and the data are represented as mean ± SD. *p < 0.05, ** *p* < 0.01, ***p < 0.001, ****p < 0.0001

Our study further aimed to identify intermediary molecules involved in SULF1’s regulation of CRC malignancy. The PPI network obtained from STRING database indicated that SULF1 could interact with ARSH (Fig. 7A). Correlation analysis using GEPIA database also suggested that SULF1 was significantly negatively correlated with ARSH (Fig. 7B). Meanwhile, survival analyses using the GEPIA database consistently showed that the mRNA expression of ARSH was positively correlated with CRC patients’ OS and DFS (Fig. 7C, D). Western blotting also revealed that ARSH was upregulated after SULF1 was knocked down in both HCT116 and SW480 cell lines (Fig. 7E). Besides, the immunoprecipitation assay further demonstrated that SULF1 interacted with ARSH (Fig. 7F). Therefore, we hypothesized that ARSH mediates the regulatory effects of SULF1 on malignant processes in CRC. To substantiate our hypothesis, we conducted proliferation, invasion, and migration experiments to further elucidate the impact of SULF1 on the malignant progression of CRC through the regulation of ARSH. As expected, the results showed that ARSH overexpression could significantly impair proliferation, migration, and invasion of CRC cells, and that effect was further amplified when both SULF1 knockdown and ARSH overexpression (Fig. 7G-K). Overall, the above results revealed that SULF1 could suppress proliferation, migration, and invasion of CRC by regulating ARSH.

### SULF1 affects CRC Cell cycle, apoptosis, and EMT by regulating ARSH via the FAK/PI3K/AKT/mTOR pathway

**Fig. 8:**
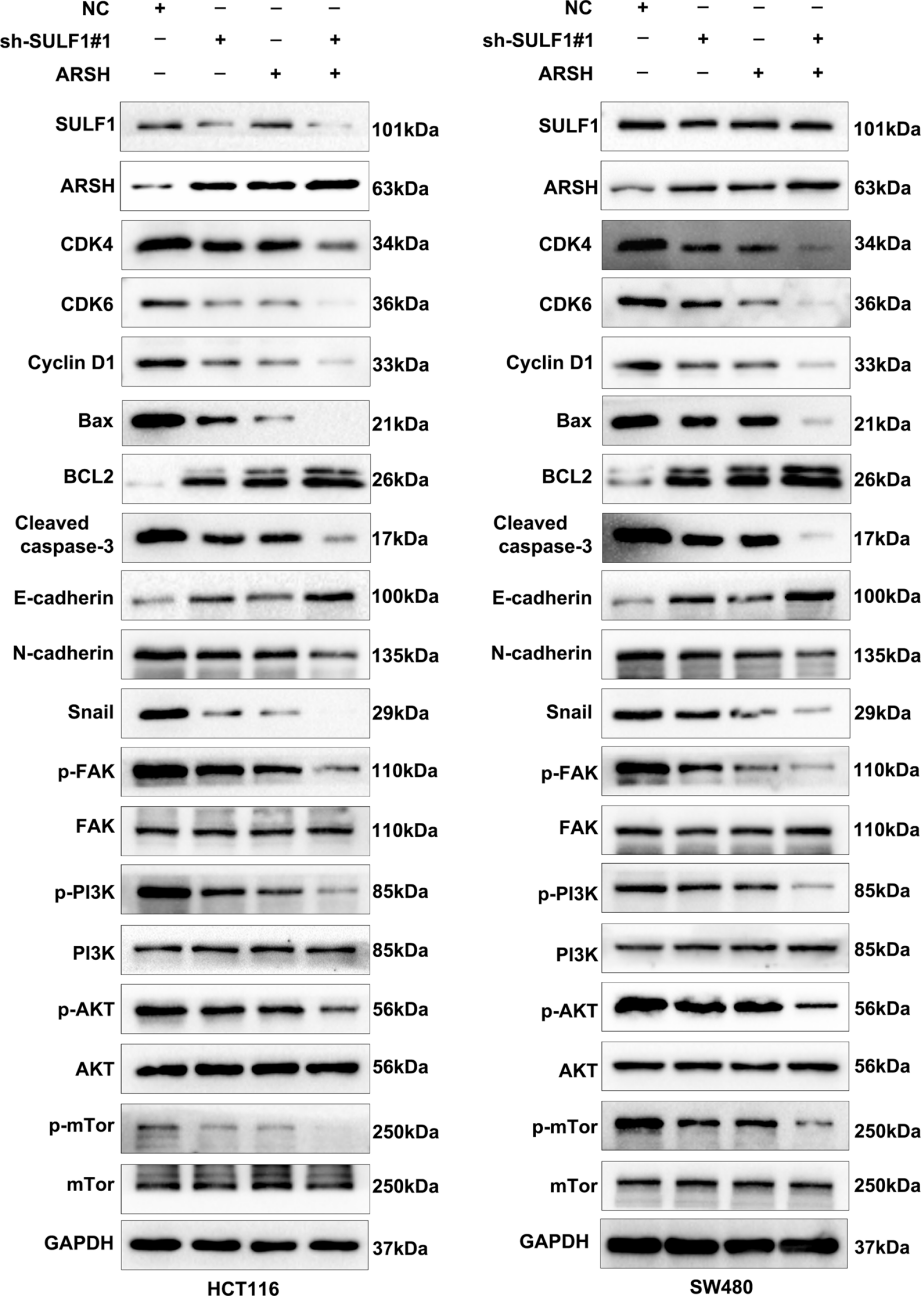
Western blotting showed that SULF1 could influence CRC cell cycle, apoptosis, and EMT by regulating ARSH via FAK/PI3K/AKT/mTOR pathway. All experiments were repeated three times and the data are represented as mean ± SD. *p < 0.05, ** *p* < 0.01, ***p < 0.001, ****p < 0.0001

To further explore whether SULF1 could affect CRC cell cycle, apoptosis, and EMT by regulating ARSH via the FAK/PI3K/AKT/mTOR signaling pathway, we performed western blotting. Increased ARSH expression resulted in upregulation of cell cycle proteins (CDK4, CDK6, Cyclin D1), pro-apoptotic proteins (Bax, Cleaved-caspase-3), and EMT markers (N-cadherin, Snail), while downregulating the anti-apoptotic protein BCL2 and the epithelial marker E-cadherin (Fig. 8). Notably, the levels of FAK, PI3K, AKT, and mTOR proteins remained unchanged upon ARSH overexpression. Interestingly, combined downregulation of SULF1 and overexpression of ARSH further enhanced these effects (Fig. 8). Taken together, these results substantiated that SULF1 knockdown could inhibit CRC cell cycle and EMT and promote CRC cell apoptosis, by regulating ARSH via the FAK/PI3K/AKT/mTOR signaling pathway.

### Knockdown of ARSH reversed the inhibitory effect of SULF1 down-regulation on malignant progression of CRC

**Fig. 9:**
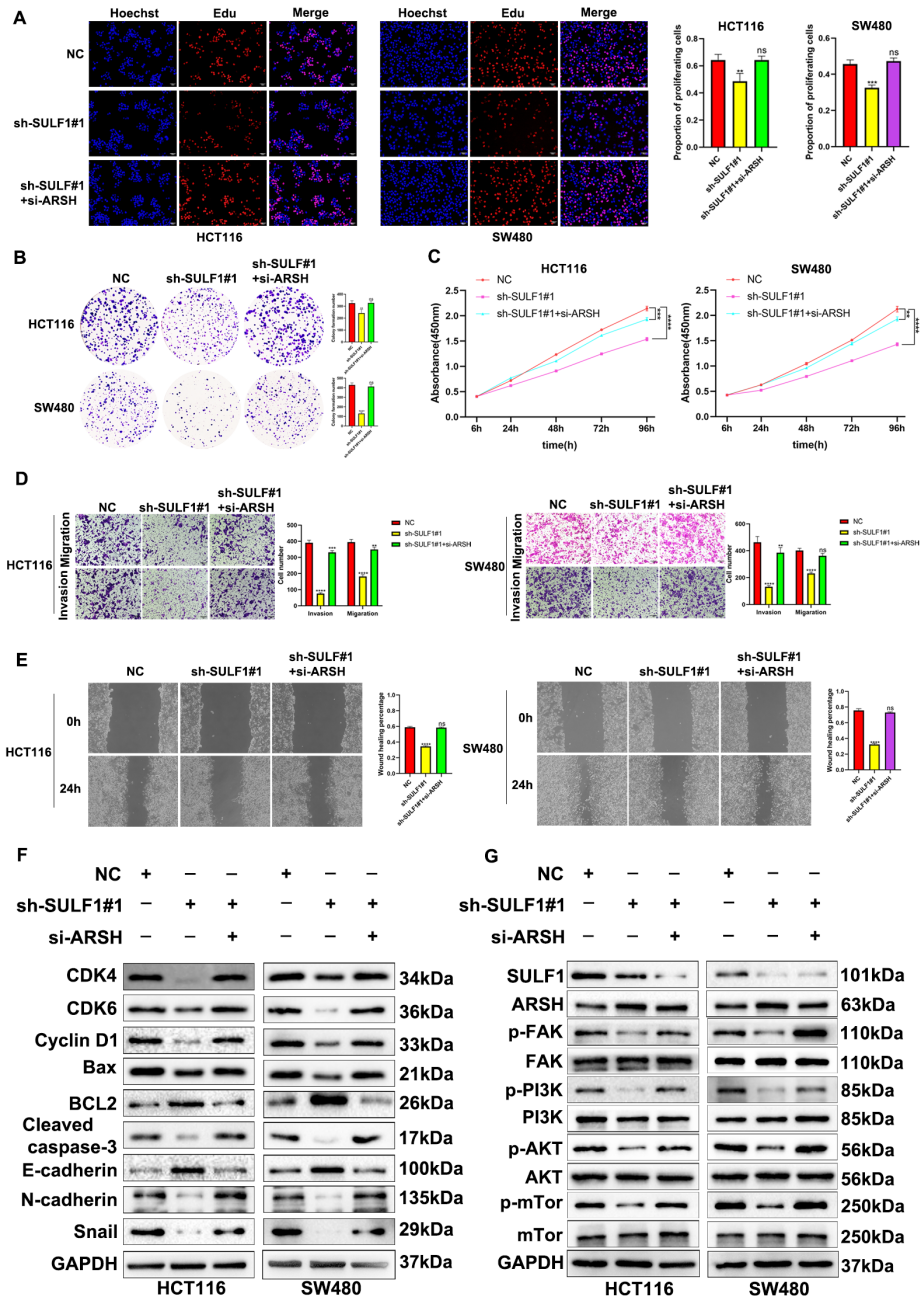
The effect of SULF1 knockdown on the proliferation, invasion and migration of HCT116 and SW480 could be recovered by eliminating ARSH. **(A-C)** EdU assay, CCK-8 assay and colony formation assay showed ARSH knockdown could eliminate the effect of SULF1 knockdown on cell proliferation. **(D)** Transwell invasion and migration assays showed that ARSH knockdown could alleviate the effect of SULF1 knockdown on cell invasion and migration. **(E)** Wound healing assay showed that ARSH knockdown could eliminate the effect of SULF1 knockdown on cell migration. All experiments were repeated three times and the data are represented as mean ± SD. *p < 0.05, ** *p* < 0.01, ***p < 0.001, ****p < 0.0001

We next assessed whether knockdown of ARSH could reverse the inhibitory effect of SULF1 down-regulation on malignant progression of CRC. EdU, CCK8, colony formation, Transwell, and wound-healing assays were performed to assess whether knockdown of ARSH could reverse the inhibitory effect of SULF1 down-regulation on proliferation, migration, and invasion ability of CRC. These results revealed that ARSH could restore the proliferative, migratory, and invasive capacities of CRC cells inhibited by SULF1 knockdown (Fig. 9A-E). In addition, Western blotting revealed that ARSH knockdown reversed the inhibitory effects of SULF1 knockdown on cell cycle progression, apoptosis, and EMT via the FAK/PI3K/AKT/mTOR pathway (Fig. 9F-G). Specifically, when both SULF1 and ARSH were downregulated, protein expression levels of cell cycle proteins (CDK4, CDK6, Cyclin D1), pro-apoptotic proteins (Bax, Cleaved-caspase-3), and EMT markers (N-cadherin, Snail) returned to control levels (NC group). Additionally, the levels of anti-apoptotic protein BCL2 and epithelial marker E-cadherin were restored. Notably, FAK, PI3K, AKT, and mTOR protein levels remained unchanged under all conditions. Overall, the above results demonstrated that knockdown of ARSH could abrogate the inhibitory effect of SULF1 down-regulation on proliferation, migration, invasion, cell cycle, apoptosis, and EMT via the FAK/PI3K/AKT/mTOR Pathway in CRC.

## Discussion

CRC is a prevalent and aggressive form of cancer, ranking third in global incidence and second in mortality rate [1]. The substantial global health burden imposed by CRC necessitates the urgent identification of effective therapeutic targets to mitigate its impact. To identify key genes potentially regulating CRC progression, we initially performed a comprehensive bioinformatics analysis utilizing four microarray datasets of CRC patients. This analysis revealed *SULF1* as a gene consistently exhibiting high expression across all four datasets.

SULF1 plays a critical role in the progression of multiple cancers. Brasil et al. [30] demonstrated that SULF1 could suppress Wnt3A-driven growth of bone metastatic prostate cancer by establishing the cancer-stroma-macrophage triculture model. Ouyang et al. [31] substantiated that the loss of SULF1 could lead to cisplatin resistance of ovarian cancer cell lines. A study by Liu et al. [32] showed that SULF1 could inhibit proliferation and invasion of esophageal squamous cell carcinoma by regulating heparin-binding growth factor signaling pathway. Besides, Lai et al. [33] reported that SULF1 suppressed growth of hepatocellular carcinoma and enhanced the effects of histone deacetylase inhibitors. Last but not least, Hur et al. [34] demonstrated that overexpression of SULF1 could promote metastasis of gastric cancer. However, no study has hitherto investigated the roles of SULF1 played in CRC. Our findings provided compelling evidence that SULF1 was highly expressed in CRC. In addition, we found that cell proliferation, invasion and migration were significantly inhibited in sh-SULF1#1 and sh-SULF1#2 groups compared with the NC group. Meanwhile, SULF1 knockdown increased cell apoptosis. Western blotting results showed that apoptosis-related protein BCL2 was up-regulated due to SULF1 knockdown, and protein expression levels of Bax and Cleaved caspase-3 were decreased. Moreover, inhibition of SULF1 can affect cell proliferation by inhibiting cell transition from G1 to S phase. Western blotting results showed that the levels of cell cycle-related proteins CDK4, CDK6 and CyclinD1 were down-regulated due to SULF1 knockdown.

ARSH is involved in the biosynthesis of hormones, regulation of cell signaling, and degradation of macromolecules [9]. Studies have shown that the ARSH gene is present in some ars operons coding for bacterial arsenic resistance/tolerance [35]. It has also been shown that ArsH is an organoarsenic oxidase that is resistant to trivalent forms of herbicide sodium methylate and poultry growth promoter Roxadone [36]. However, ARSH has been understudied in human tumors, and its role in CRC remains largely unclear. STRING analysis indicated that SULF1 could interact with ARSH, and immunoprecipitation confirmed the above hypothesis. The results showed that ARSH negatively regulated by SULF1 inhibited the proliferation, invasion and migration of CRC cells. The co-transfection of sh-SULF1 and ARSH further inhibited the proliferation, invasion and migration of CRC cells. While the FAK/PI3K/AKT/mTOR pathway appears to be a key mediator of SULF1’s effects in CRC, our findings prompt further exploration of the potential involvement of ARSH. To investigate this possibility, we constructed an ARSH knockdown cell line. The results showed that ARSH knockdown could restore the inhibitory effect of SULF1 knockdown on the proliferation, invasion and migration of CRC cells.

We next conducted GO and KEGG enrichment analysis to explore the potential mechanisms by which SULF1 influences CRC progression. GO and KEGG results indicated a potential link between SULF1 and the abnormal phosphorylation of FAK. PI3K, AKT and mTOR are important downstream genes of FAK [37]. Western blotting results showed that p-FAK activity was significantly inhibited by SULF1 knockdown, and this effect was further enhanced after ARSH was upregulated. In addition, knocking down ARSH yielded the opposite effect on p-FAK as SULF1. PI3K/AKT/mTOR is a component of an important signaling pathway in CRC and a downstream gene of FAK, which is activated by FAK in the phosphorylated state. Therefore, we continued to explore the expression levels of p-PI3K, p-AKT and p-mTOR in different groups of CRC cells, and these results were consistent with the above results.

EMT is a well-documented process by which epithelial tumor cells acquire a more motile and invasive phenotype, enhancing their aggressive potential [38]. During EMT, epithelial markers such as E-cadherin decrease in expression, while mesenchymal markers like N-cadherin and vimentin increase [39]. Many studies have shown that the FAK signaling pathway promotes EMT process of cells in tumors [40, 41]. Therefore, we explored whether SULF1 influences EMT pathways in CRC through FAK signaling. Western blotting analysis revealed that knockdown of SULF1 (sh-SULF1#1 and sh-SULF1#2) resulted in decreased expression of p-FAK and mesenchymal markers (N-cadherin and vimentin), while E-cadherin expression was upregulated. Furthermore, the protein levels of p-PI3K, p-AKT, and p-mTOR mirrored the changes observed in p-FAK, suggesting a potential link between SULF1 and this signaling cascade. Interestingly, ARSH expression was elevated in the sh-SULF1#1 group, coinciding with a further modulation of the aforementioned protein markers. To delve deeper into the role of ARSH, we next investigated the effect of ARSH knockdown on the sh-SULF1#1 group, which revealed that ARSH knockdown could reduce the inhibitory effect of SULF1 knockdown on EMT and FAK signaling.

Finally, CRC cells from the NC group or sh-SULF1#1 group were injected subcutaneously into nude mice to study the effect of SULF1 in vivo. The results showed that SULF1 knockdown could significantly inhibit the growth rate of tumors, indicating that SULF1 knockdown could inhibit tumor growth in vivo.

Our study has established the regulatory role of SULF1 in CRC progression via the ARSH/FAK/PI3K/AKT/mTOR pathway, supported by bioinformatics analysis, in vitro experiments, and an in vivo tumor model. However, several limitations warrant further investigation. First, we focused on the inhibitory effect of SULF1 knockdown on CRC malignancy. Future studies should explore whether SULF1 overexpression promotes CRC progression. Second, while the subcutaneous tumor model confirmed the suppressive effect of SULF1 knockdown on tumor growth, the impact on metastasis remains unexamined. Investigating whether SULF1 knockdown affects lung or liver metastasis would be valuable. Third, we validated the interaction between SULF1 and ARSH, including its regulation of ARSH expression. However, the specific mechanism underlying this regulation requires further elucidation.

In conclusion, our in vitro and in vivo studies reveal that SULF1 negatively regulates ARSH expression through the FAK/PI3K/AKT/mTOR pathway. Our study provides hitherto undocumented evidence of SULF1 as a key player in CRC progression. SULF1 knockdown demonstrably inhibits CRC cell proliferation by inducing apoptosis and cell cycle arrest, while also suppressing invasion through EMT inhibition. These findings collectively suggest that SULF1 represents a promising novel therapeutic target for CRC.

### Electronic supplementary material

Below is the link to the electronic supplementary material.

Supplementary Material 1

Supplementary Material 2

Supplementary Material 3

Supplementary Material 4

Supplementary Material 5

Supplementary Material 6

Supplementary Material 7

Supplementary Material 8

Supplementary Material 9

Supplementary Material 10

Supplementary Material 11

Supplementary Material 12

Supplementary Material 13

## Data Availability

The datasets are available from TCGA (https://portal.gdc.cancer.gov/) and Gene Expression Omnibus (https://www.ncbi.nlm.nih.gov/geo/).
