# Supplementary figures and images for: SULF1 regulates malignant progression of colorectal cancer by modulating ARSH via FAK/PI3K/AKT/mTOR signaling
Source: Cancer Cell Int. 2024 Jun 6;24:201. doi: 10.1186/s12935-024-03383-5 (PMC11157809; doi:10.1186/s12935-024-03383-5)

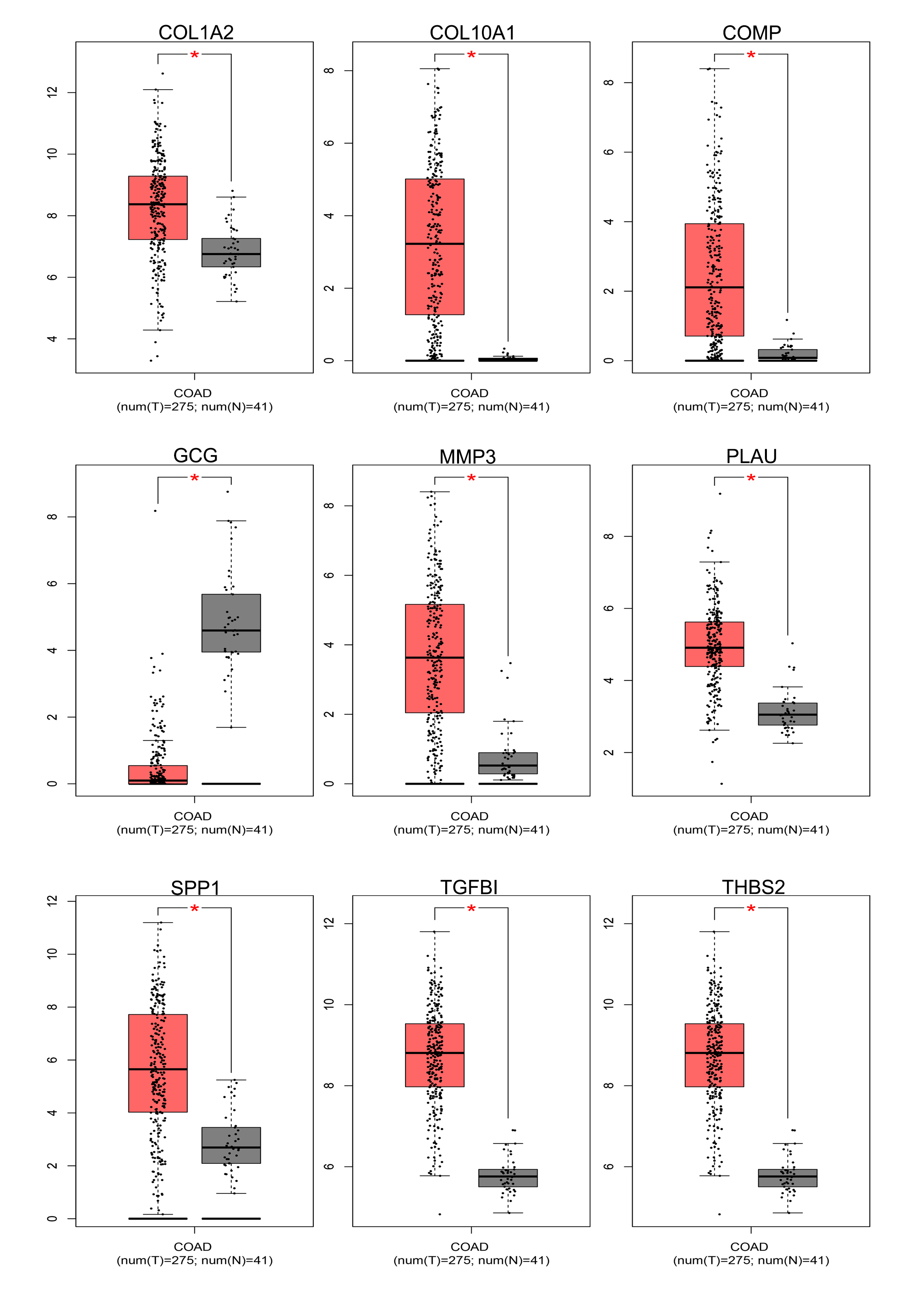

Supplement: Supplementary file 1 — Supplementary Material 1 [file 12935_2024_3383_MOESM1_ESM.tif]

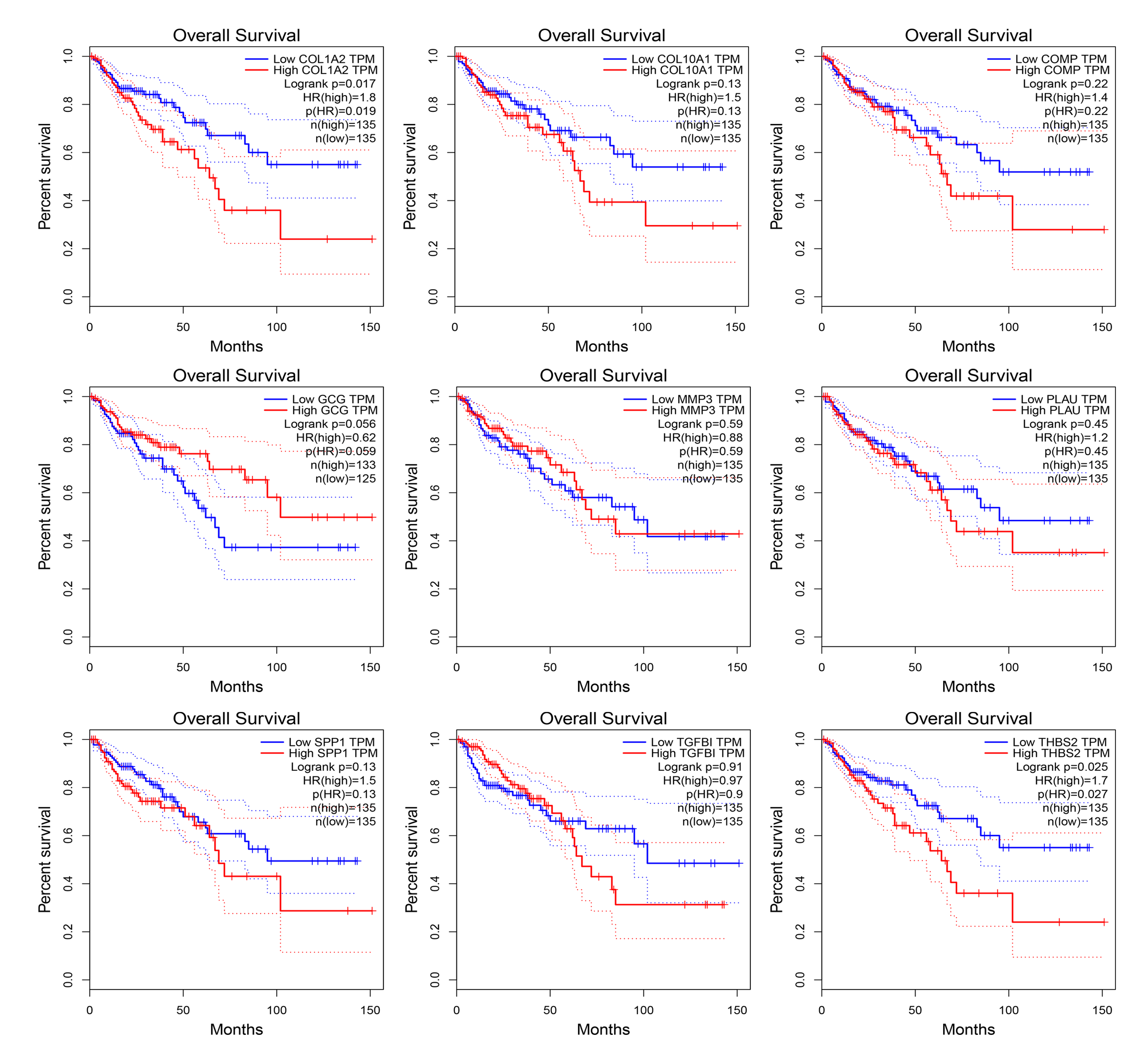

Supplement: Supplementary file 2 — Supplementary Material 2 [file 12935_2024_3383_MOESM2_ESM.tif]

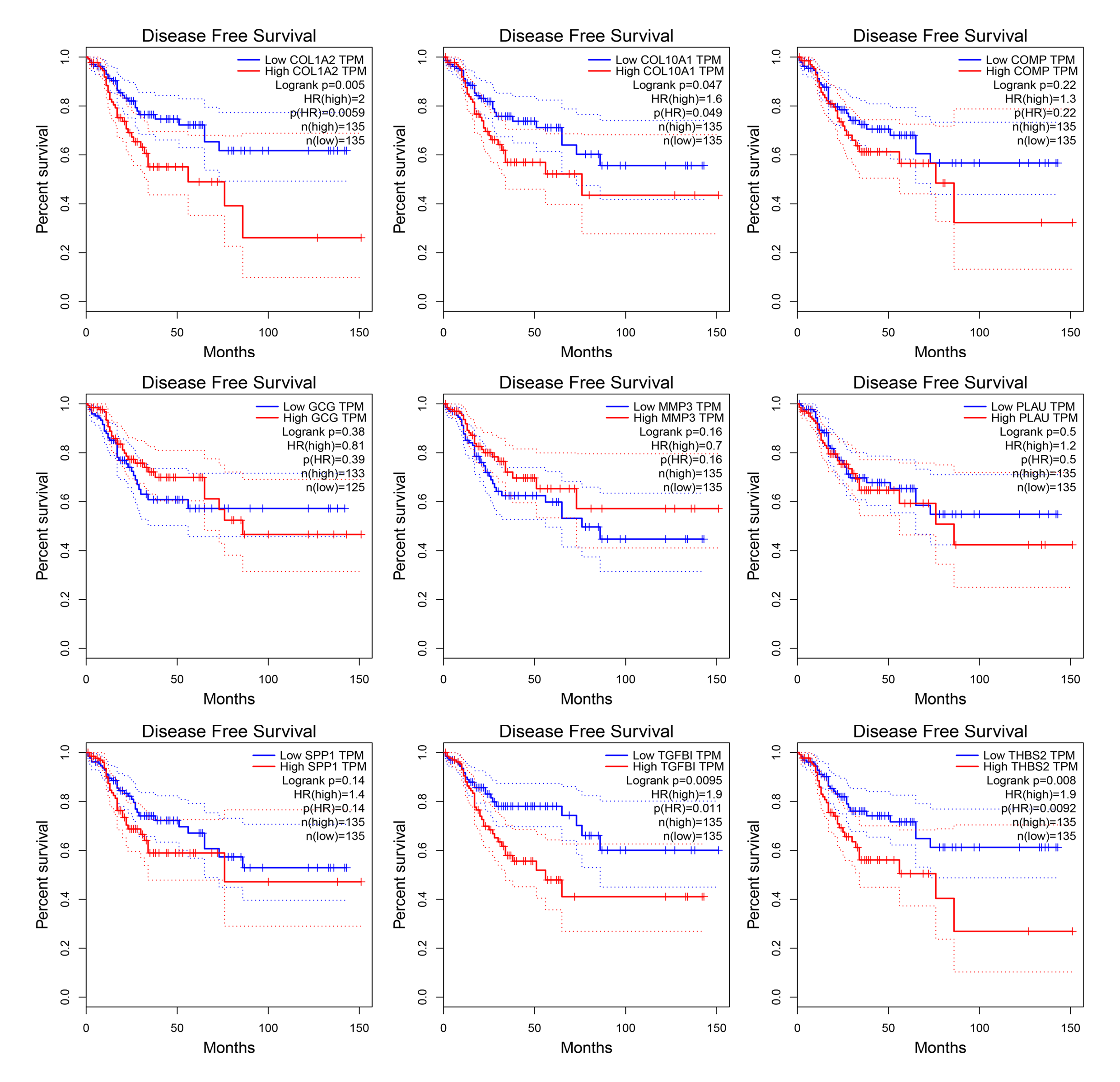

Supplement: Supplementary file 3 — Supplementary Material 3 [file 12935_2024_3383_MOESM3_ESM.tif]
